# Is There a Role for Machine Learning in Liquid Biopsy for Brain Tumors? A Systematic Review

**DOI:** 10.3390/ijms24119723

**Published:** 2023-06-03

**Authors:** Grazia Menna, Giacomo Piaser Guerrato, Lal Bilgin, Giovanni Maria Ceccarelli, Alessandro Olivi, Giuseppe Maria Della Pepa

**Affiliations:** Institute of Neurosurgery, Fondazione Policlinico Universitario A. Gemelli IRCCS, Catholic University of Rome, 00168 Roma, Italygiacomo.piaserguerrato@gmail.com (G.P.G.); gianmariacecca@gmail.com (G.M.C.); alessandro.olivi@policlinicogemelli.it (A.O.)

**Keywords:** machine learning, liquid biopsy, brain tumor

## Abstract

The paucity of studies available in the literature on brain tumors demonstrates that liquid biopsy (LB) is not currently applied for central nervous system (CNS) cancers. The purpose of this systematic review focused on the application of machine learning (ML) to LB for brain tumors to provide practical guidance for neurosurgeons to understand the state-of-the-art practices and open challenges. The herein presented study was conducted in accordance with the PRISMA-P (preferred reporting items for systematic review and meta-analysis protocols) guidelines. An online literature search was launched on PubMed/Medline, Scopus, and Web of Science databases using the following query: “((Liquid biopsy) AND (Glioblastoma OR Brain tumor) AND (Machine learning OR Artificial Intelligence))”. The last database search was conducted in April 2023. Upon the full-text review, 14 articles were included in the study. These were then divided into two subgroups: those dealing with applications of machine learning to liquid biopsy in the field of brain tumors, which is the main aim of this review (n = 8); and those dealing with applications of machine learning to liquid biopsy in the diagnosis of other tumors (n = 6). Although studies on the application of ML to LB in the field of brain tumors are still in their infancy, the rapid development of new techniques, as evidenced by the increase in publications on the subject in the past two years, may in the future allow for rapid, accurate, and noninvasive analysis of tumor data. Thus making it possible to identify key features in the LB samples that are associated with the presence of a brain tumor. These features could then be used by doctors for disease monitoring and treatment planning.

## 1. Introduction

Despite a substantial increase in publications in recent years, liquid biopsy (LB) is not routinely used yet in cancer diagnostics and tumor monitoring. In contrast to direct biopsy, LB uses body fluids collected distant to the primary tumor site, such as venous blood or cerebrospinal fluid (CSF). LB may miss a relevant part of a tumor, which tissue biopsy may detect and vice versa; however, LB may be more representative of its more migratory and aggressive counterpart. Tumor-derived material may be in either free-form (circulating tumor nucleic acids and circulating tumor cells (CTCs)) or within membrane-bound vesicles (microvesicles (MVs) and exosomes (EXs)). More recently, the blood platelets, a neglected source of tumor cell information, showed their vast potential in liquid biopsy as tumor-educated platelets (TEPs) [1].

Against this background, intrinsic brain tumors represent an additionally challenging group of tumors for several reasons such as their low incidence and cost-effectiveness of early screening, the lack of evidence for premature treatment options, and the presence of blood–brain barrier (BBB) as a potential suppressor of migrating tumor cells and their poor ability to metastasize through blood [2,3,4]. One of the main challenges for integrating brain tumor LB into clinical routine remains in the evaluation of reliable standards and in the increase of the rather low and variable sensitivity that is currently at around 10–60%, since the shedding of tumor DNA into CSF does not appear to be a universal property of diffuse glioma, even in previously treated patients. Furthermore, there are still insufficient data on the influence of tumor type (glioblastoma vs. IDH-mut astrocytoma vs. lower grade gliomas), location, extent of the BBB disruption, and disease stage on sensitivity, specificity, and clinical utility of individual liquid biopsy biomarkers and their combination, in addition to the best modality of cerebrospinal fluid (CSF) collection (either lumbar or cisternal CSF) [5,6].

As already mentioned, one of the main drawbacks of LB is the proper clinical interpretation of the increasingly large sets of data that these technologies generate. The data sets of cancer LB are large and complex. Therefore, it is difficult to use traditional statistical methods. Machine learning (ML) algorithms can automatically analyze and identify patterns from such complex and heterogenous data. So far, an increasing number of original papers as well as many reviews have outlined the challenges in finding and applying different ML methods to improve the LB detection of the most common tumors (breast, prostate, and colorectal carcinomas). However, limited machine learning expertise in the LB field has kept the discipline from fully leveraging these tools and risks improper analyses and irreproducible results [7].

Diagnosis of brain tumors is often a challenge because of the need to obtain tissue samples, and this often involves invasive procedures. Therefore, the use of LB may be a noninvasive and rapid tool for tumor profiling. The repeatability of the analysis could also allow for easy monitoring of the disease progression. Another technology that could help in this regard is the use of radiomics systems, which allow, when using ML algorithms, for the analysis of the main features of brain neoformations in a noninvasive way [8,9].

The paucity of studies regarding the applications of LB to central nervous system (CNS) tumors in the brain tumor literature demonstrates that this field of application is still an under-explored area in biomedical research. In fact, liquid biopsy represents a recently introduced technology; features found in the systemic circulation associated with the presence of a tumor, and particularly brain tumors, are still being defined and classified. In this area, the use of ML techniques that are also only recently beginning to be an active part of clinical practice, can be of support in identifying patterns that are useful to the clinician. Our literature search returned studies conducted mainly in recent years, an aspect indicative of a new way of approaching cancer diagnosis and monitoring that has recently emerged and is rapidly evolving.

Therefore, the purpose of this systematic review focused on the application of ML to LB for brain tumors to provide practical guidance for neurosurgeons to understand the state-of-the-art practices and open challenges.

## 2. Materials and Methods

### 2.1. Review Question

The herein presented study was conducted in accordance with the PRISMA-P (preferred reporting items for systematic review and meta-analysis protocols) guidelines [10]. An online literature search was launched on PubMed/Medline, Scopus and Web of Science databases using the following query: “((Liquid biopsy) AND (Glioblastoma OR Brain tumor) AND (Machine learning OR Artificial Intelligence))”. The last database search was conducted in April 2023.

### 2.2. Inclusion and Exclusion Criteria

Two authors (G.P.G. and L.B.) independently conducted the abstract screening for eligibility. Any discordance was solved by consensus with a third author (G.M.). No restrictions were made based on the date of publication. The inclusion criteria included studies that dealt with applications of LB for brain tumor diagnosis and monitoring; in addition, these had to make use of ML algorithms for data analysis to facilitate the development of models that would allow for easy tumor identification and differentiation from noncancerous conditions. The exclusion criteria were as follows: studies published in languages other than English, reviews, editorials, protocols, and studies involving applications of LB but without the use of ML or ML not used for LB analysis. A systematic abstract screening of the references (forward search) was performed to identify additional records. Each report was further analyzed in steps. The following key aspects were identified: the number of samples or the number of patients included in the studies, type of medium and type of tumor, and type of machine learning algorithm used.

## 3. Results

The search of the literature yielded a total of 55 results. Duplicate records were then removed (n = 24). Thirty-one records were screened and four records were excluded through title and abstract screening; 27 reports were sought for retrieval and four studies were not accessible and so discarded. Twenty-three reports were found to be relevant to our research question and assessed for eligibility. Nine records were excluded as they did not meet the inclusion criteria. In one case, the machine learning approach was applied to imaging [11], in other cases, machine learning was not used in the data analysis phase [12,13,14], and five additional studies were excluded due to the presence of keywords but they did not match our previously stated inclusion criteria [15,16,17,18,19].

**Figure 1:**
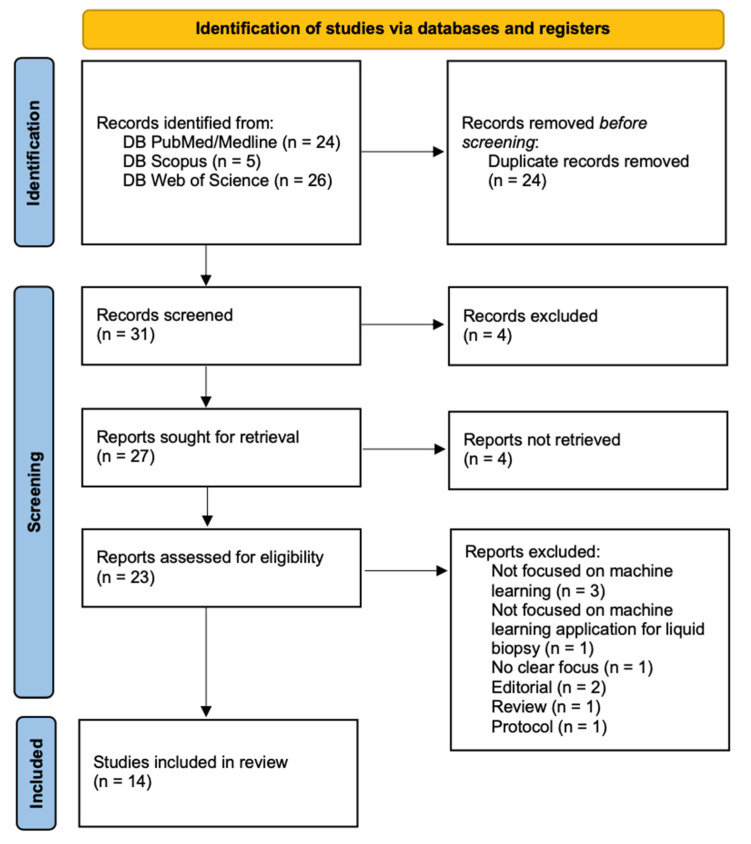
PRISMA flow diagram.

Upon the full-text review, 14 articles were included in the review (Figure 1). The studies were then divided into two subgroups: those dealing with applications of machine learning to liquid biopsy in the field of brain tumors, which was the main aim of this review (Table 1, n = 8); and those dealing with applications of machine learning to liquid biopsy in the diagnosis of other tumors (Table 2, n = 6). As the number of studies exclusively concerning brain tumors was low, this second group was included to emphasize how ML applications may be a valuable aid in cancer diagnosis, with possible future applications to brain tumors as well. The inclusion and exclusion criteria used were the same in both groups, and the division was made referring only to the tumor types studied.

## 4. Discussion

As already discussed, many studies have used the LB approaches to improve cancer screening, diagnosis, and prognosis, as well as the classification of more heterogeneous entities, the assessment of treatment response, and the detection of treatment-resistant subclones. The main advantages of LB are that they are non-invasive, repeatable, and real-time assessable [2,3,4,5,6].

Intrinsic brain tumors represent a challenging group of tumors in which the use of liquid biopsy can be routinely applied. Only sparse and early evidence has been published in the literature reporting that different phases of gliomagenesis are characterized by different secreted proteomics biomarkers. There is a role of LB in establishing a diagnosis when tissue biopsy is not feasible due to the risk of an excessive morbidity, such as in deep-seated or multicentric lesions, or in the presence of advanced age and a burden of comorbidities. However, this is not to say that there is no potential for LB in gliomas at other stages such as the monitoring of treatment outcomes and prognostic stratification of affected patients. The level of an ideal, hypothetical biomarker should be elevated at the time of diagnostic imaging, then drop significantly after surgical removal of the tumor, and remain low during additional treatment, thus helping to differentiate progression from pseudo-progression and increasing the number of cases eligible for potentially curative options or to receive more successful therapies. This is truer today since the 2021 WHO CNS tumor classification requires a brain tumor characterization not only based on their histology but also, independently, based on their molecular features [34].

As already stated, the paucity of work present in the literature on brain tumors demonstrates that liquid biopsy (LB) is not currently applied for CNS cancers. The purpose of this systematic review is to review the application of ML to LB for brain tumors first through the analysis of the choice of sample- and tumor-derived material, then carefully reviewing the choice of ML and their real advantage in terms of improving diagnostic accuracy, and finally comparing ML applications in brain tumor detection and follow-up to other types of cancers.

### 4.1. Choice of Sample

The sampling of proximal fluids has offered valuable insight into various brain tumors. To perform a liquid biopsy, samples taken from blood (serum, plasma, buffy coat, and whole blood), urine, CSF, and others were used.

Blood and CSF are the most relevant proximal fluids in neuro-oncology, with CSF ideally considered as the best LB source for brain tumors, owing to its direct contact with the tumor microenvironment and limited obstruction by the BBB. In routine clinical practice, CSF sampling is central to the management of central nervous system lymphoma (CNSL), it is routinely used for prognostication of medulloblastoma and germ cell tumors; in GBM and metastases, it is not part of routine clinical practice [24].

While analysis of tumor-derived material in CSF has improved detection frequencies, this biofluid is both difficult to collect and associated with significant discomfort for the patient. Therefore, it is less likely to be considered as a viable approach for longitudinal sampling going forward. The non-invasive nature of plasma and urine may permit more regular and less restrictive monitoring for GBM patients than CSF sampling [20].

The most reported sample was blood/serum. Patient serum-derived LB can be a helpful tool for diagnostic purposes. A TR-FTIR spectroscopy-based liquid biopsy test correctly determined who among 385 patients with suspected brain cancer and referred from primary care for brain imaging had cancer. Infrared spectroscopy is a phenotypic method that quantifies the absorption of mid-infrared light resulting in a specific FTIR spectrum that reflects the overall composition of the sample. Vibrational spectroscopy techniques, such as ATR-FTIR, require only small amounts of material (µL range). This rapid (15 min is typical per patient sample), low-cost test can integrate with a clinical assessment in primary care to help identify which patients to prioritize for diagnostic imaging; and achieve earlier cancer detection and diagnosis [22].

Furthermore, examination of the molecular content of sEVs by Raman spectroscopy has been demonstrated as a promising tool in diagnosing CNS tumors [26].

Another study by Tsvetkov et al. applied the principle of differential scanning calorimetry (DSC) to serum-derived LB. DSC is a biophysical method used to study the thermal denaturation of proteins, where the observed differences in denaturation profiles of biofluids from healthy controls and patients could be explained by changes in thermostability of the most abundant proteins or by the change in their relative concentrations. Independent of the underlying molecular mechanisms resulting in this change, the denaturation profile itself can therefore be used as a biomarker. They used NanoDSF, which was originally designed to study protein thermostability and was based on the modifications of the intrinsic fluorescence of the macromolecules upon their thermal denaturation. The tool was employed to analyze the plasma of patients affected by glioma and to develop a novel AI-based method to automatically distinguish the denaturation profiles of affected patients from healthy controls [23].

Furthermore, LB was found to be effective in a longitudinal study of glioma patients, as demonstrated by Morokoff et al. who identified a 9-gene miRNA signature that could distinguish between glioma and healthy controls with 99.8% accuracy. Two miRNAs, miR-223 and miR-320e, best demonstrated dynamic changes that correlated closely with tumor volume in LGG and GBM, respectively. Importantly, miRNA levels did not increase in two cases of pseudo-progression [25].

In addition to gliomas, blood-derived LB can be useful also in challenging sellar disease cases, such as rare primary or secondary sellar tumors, or non-neoplastic diseases that may be misdiagnosed as PitNETs, and that could benefit from a presurgical and noninvasive diagnostic approach to better guide the appropriate management, suggesting that serum or plasma cfDNA from patients with PitNETs contain methylation fingerprints specifically related to these tumors [21].

### 4.2. Choice of Tumor-Derived Material

The analyzed tumor-derived material later used in computational analysis were: tumor-educated platelets (TEPs), cell-free DNA (cfDNA), circulating tumor DNA (ctDNA), miRNA, extracellular vesicles and particles (EVPs), and serum-derived small extracellular vesicles (sEVs).

Tumor-derived material may be in either free-form (circulating tumor nucleic acids and circulating tumor cells (CTCs)) or within membrane-bound vesicles (microvesicles (MVs) and exosomes (EXs)). CTCs are cells derived from a tumor that enter the bloodstream and other body fluids. Applying the seed-and-soil hypothesis, they may retain the ability to spread (seed), but they do not necessarily find the right target tissue or endothelium (soil). Circulating tumor DNA (ctDNA) represents only a fraction of the total cell-free DNA (cfDNA), but is typically shorter than the latter, thus allowing for an easier selection. MicroRNAs (miRNA, miR) are noncoding RNA molecules involved in the regulation of the stability and translation of mRNA. One of their main drawbacks is the difficulty in distinguishing tumor patients not only from noncancer individuals, but also from patients with other diseases via miRNA.

There are two types of EVs and they differ mainly in their size: exosomes (30–150 nm diameter) and microvesicles (MVs) (150–1000/nm). No standard protocols exist to specifically isolate and separate exosomes from MVs. There is evidence that EVs secreted by tumor cells may be taken up by neighboring and distant cells, resulting in intercellular communication and microenvironment modulation.

More recently thrombocytes, a neglected biosource of tumor cell information, showed their vast potential in liquid biopsy. Platelets are small enucleated cellular fragments that are 2–4 µm in diameter and derived from megakaryocyte cells in the bone marrow. Since platelets were first described, they have been discovered to have a pivotal biological role at several stages of malignant disease. Bidirectional interaction between cancer cells and platelets is evidently reciprocal, as carefully summarized by Yu et al. in their review [35,36].

In their pioneering study, Sol et al. showed how TEP-derived RNA signatures allowed for the separation of patients with glioblastoma from those with metastases to the brain with 92% accuracy, and could differentiate glioblastoma from other brain lesions. Furthermore, TEP RNA profiles of patients with glioblastoma are different from those of asymptomatic healthy controls, and TEP RNA signatures were correlated to tumor volume and tumor recurrence and may have facilitated the discrimination of true tumor progression from false positive progression [27].

### 4.3. Machine Learning Application

What all the previously cited studies have in common is the use of ML algorithms to improve their diagnostic accuracy.

The machine learning algorithms used are: random forest (RM), support vector machine (SVM), particle-swarm optimization (PSO), principal component analysis (PCA), adaptive boosting (AdaBoost), partial least squares-discriminant analysis (PLS-DA), neural networks, decision trees (DT), gradient boosted decision trees, k-nearest neighbor (k-NN), and linear regression (LR) in combination with other data pre-processing and data prediction algorithms.

The most commonly used computational analysis environments were R (R Project for Statistical Computing) and Python (Python Software Foundation, Wilmington, DE, USA).

The main advantage of machine learning algorithms is the ability to analyze a large amount of data samples and to build models considering, even simultaneously, different features. Comparison between samples of healthy and affected subjects is particularly helpful in this process. Basically, ML allows for the identification of patters, characteristics, and genes, which in a conventional situation one would already need to know—these once “discovered” during the training phase can be used in clinical practice. In the context of liquid biopsy, the difficulties in identifying key features associated with the presence of a tumor could thus be overcome using this approach.

Performing a liquid biopsy and analyzing it based on models built with these algorithms could enable simple and noninvasive cancer diagnosis and monitoring. furthermore, considering that at present, correct identification and classification of a brain tumor often requires invasive biopsy with all of the associated risks, these new tools are particularly promising.

In this context, the algorithms used have been shown to be able to detect specific patterns that can enable the identification of tumor-associated features.

It has been noted that ctDNA obtained from urine samples of patients with glioma, is more fragmented than that of healthy individuals [20]. In two other studies, serum spectroscopy approaches were used, and machine learning enabled the detection of spectral variations between healthy and tumor-affected patients [22,26]. The use of plasma denaturation profiles, CSF proteomic signature, and serum miRNA signature have also proven to be a valid system of tumor detection [23,24,25,26,27]. In another case, tumor-instructed platelets (TEPs) were studied, algorithms were used to select a panel of spliced RNA biomarkers, and the scores obtained represented tumor behavior, and could thus be used to distinguish false-positive from true progression [27]. Machine learning was also used to develop a score that could distinguish PitNET from non-PitNET conditions [21].

### 4.4. Application of Machine Learning to Other Types of Cancer

We also analyzed studies dealing with the application of machine learning algorithms in other types of cancers. The use of these tools has proven to be a promising system as well in these cases [29,30,31].

In two studies, blood gene expression profiles and extracellular microRNA profiles were obtained from the Gene Expression Omnibus [30,32]. The latter can be defined as an international public repository that archives and freely distributes microarray, next-generation sequencing, and other forms of high-throughput functional genomics data submitted by the research community.

Analysis of extracellular vesicle and particle (EVP) content obtained from tissue and plasma explants, in combination with machine learning algorithms, has identified panels that can be used to classify tumors of unknown primary origin [30]. TEPs have also been studied, and algorithms were used to identify RNA biomarkers, key genes, and patterns for cancer detection and monitoring [29,30,31].

The use of these algorithms allowed for the differentiation between samples from healthy individuals or those with noncancerous conditions and those with tumors. More work needs to be carried out to identify unique patterns associated with a specific tumor to enable true liquid biopsy.

## 5. Conclusions

In this review, we summarized the reasons why machine learning can have a role in LB for brain cancer: (1) early detection, since its more effective than traditional statistical methods in identifying the early signs of brain cancer by analyzing the genetic and proteomic markers present in liquid biopsies; (2) precision medicine, since it can rapidly help predict the effectiveness of various therapies by analyzing the molecular signature of tumors; (3) faster results: ML algorithms can analyze LB results quickly and precisely providing doctors with actionable insights that can help inform treatment decisions.

Despite the excellent premises, the paucity of studies and possible selection bias due to the heterogeneity of techniques highlight the need for further and extensive investigation of the use of LB and ML in the context of brain tumors.

## Figures and Tables

**Table 1:** Table summarizing the papers focused on ML applications to LB for brain tumors.

Author	Year	Medium	Tumor-Derived Material	N° pt/Samples	Type of Tumor	Machine Learning Algorithms	Outcome
Mouliere, Florent, et al. [20]	2021	CSF, plasma, urine	cfDNA	88 patients	HGG (GBM, anaplastic oligodendroglioma), LGG (diffuse astrocytoma, oligodendroglioma, pilocytic astrocytoma), IDH wild type and IDH mutant	LR + random forest + support vector machine + binomial generalized linear model with elastic-net regulation	cfDNA in urine of glioma patients was significantly more fragmented compared to urine from patients with non-malignant brain disorders and healthy individuals (*p* = 5.2 × 10^−9^). Machine learning models integrating fragment length could differentiate urine samples from glioma patients (AUC = 0.80–0.91).
Herrgott, Grayson A., et al. [21]	2022	Serum, plasma	cfDNA	100 samples [59 serum + 41 plasma]	PitNET, non-PitNET, other pituitary diseases (OPD)	Random forest	Using LB-derived PitNETs-specific signatures as input to develop machine learning predictive models, a score was generated that could distinguish PitNET from non-PitNET conditions, including sellar tumors and non-neoplastic pituitary diseases, with accuracies above ~93% in independent cohort sets.
Theakstone, Ashton G., et al. [22]	2021	Serum	ATR FTIR	177 patients	HGG (glioblastoma (GBM) or anaplastic astrocytoma), LGG (astrocytoma, oligoastrocytoma and oligodendroglioma)	Random forest + partial least squares-discriminant analysis + support vector machine	The machine learning techniques are required to identify any spectral variations between the samples. Sensitivities, specificities, and balanced accuracies were all greater than 88%, the area under the curve (AUC) was 0.98, and cancer patients with tumor volumes as small as 0.2 cm^3^ were correctly identified.
Tsvetkov, Philipp O., et al. [23]	2021	Plasma	Denaturation profiles [15° to 95°—plasma profiling with nanoDSF]	147 patients	22 patients (26%) with 1p/19q codeleted *IDH* mutated oligodendroglioma, 25 patients (31%) with *IDH* mutated astrocytoma, 37 patients (43%) with *IDH* wild-type astrocytoma (including 19 *IDHwt* glioblastomas)	LR + support vector machine (SVM) + neural networks + random forest + adaptive boosting (AdaBoost) + false-negative focusing SVM	Denaturation profiles can be automatically distinguished with the help of machine learning algorithms with 92% accuracy. A high throughput workflow is also proposed that can be applied to any type of tumor.
Mikolajewicz, Nicholas, et al. [24]	2022	CSF	CSF proteomics [shotgun proteomics]	73 samples	Glioblastoma (GBM), brain metastases (BM), primary central nervous system lymphoma (CNSL)	LR machine learning classifier framework + Caret	A logistic regression (LR) machine learning classifier framework was used to evaluate if proteomic signatures can detect malignancy using CSF proteomics. Proteomic-based LR classifiers identified malignancy with a median AUROC of 0.94 (95% CI, 0.85–1.0), and this estimate ranged from 0.95 to 1.0 when evaluated for single neoplastic entities, demonstrating minimal neoplasm-specific bias. Machine learning approach was then used to nominate individual proteins for each type of brain neoplasm for clinical application.
Morokoff, Andrew, et al. [25]	2020	Serum	Serum miRNA profiling [exosome and non-exosome]	108 patients	Gliomas	Random forest with Monte-Carlo based validation approach	Machine learning was used to generate a model with a minimum number of features (miRNAs). Following subsequent analysis, a 9-gene miRNA signature was identified that could distinguish between glioma and healthy controls with 99.8% accuracy. Two miRNAs, miR-223 and miR-320e, best demonstrated dynamic changes that correlated closely with tumor volume in LGG and GBM, respectively. Importantly, miRNA levels did not increase in two cases of pseudo-progression.
Bukva, Matyas, et al. [26]	2021	Serum	sEVs [Raman spectroscopy]	138 samples	Glioblastoma multiforme, non-small-cell lung cancer brain metastasis, meningioma and lumbar disc herniation	Principal component analysis–support vector machine + FreeViz	Machine learning algorithm was performed on the Raman spectra for pairwise classifications. The groups compared were distinguishable with 82.9–92.5% CA, 80–95% sensitivity, and 80–90% specificity. AUC scores of 0.82–0.9.
Sol, Nik, et al. [27]	2022	Blood	TEPs RNA	805 samples	Glioblastoma comparison vs. multiple sclerosis and brain metastasis patients + glioblastoma vs. asymptomatic healthy controls	Particle-swarm intelligence-enhanced support vector machine algorithm thromboSeq (conventional and variant)	Training samples were employed to select a TEP tumor score of individual glioblastoma patients representing tumor behavior that could be used to distinguish false positive progression from true progression (validation series, n = 20; accuracy, 85%; AUC, 0.86 [95% CI, 0.70–1.00; *p* < 0.012]).

**Table 2:** Table summarizing the papers focused on ML applications to LB for other cancers.

Author	Year	Medium	Tumor-Derived Material	N° pt/Samples	Type of Tumor	Machine Learning Algorithms	Outcome
Hoshino, Ayuko, et al. [28]	2020	Various samples	EVPs	497 samples [426 human + 71 murine]	Various cancers	Random forest + PAM + Caret	Machine learning classification of plasma-derived EVP cargo, including immunoglobulins, revealed 95% sensitivity and 90% specificity in detecting cancer. A panel of tumor-type-specific EVP proteins in tissue explants and plasma that can classify tumors of unknown primary origin was defined.
Best, Myron G., et al. [29]	2017	Platelets (blood)	TEPs RNA	779 samples	NSCLC	Particle-swarm intelligence-enhanced support vector machine algorithm thromboSeq	Particle-swarm optimization (PSO)-enhanced algorithms enable efficient selection of RNA biomarker panels from platelet RNA-sequencing libraries. This resulted in accurate TEP-based detection of early- and late-stage non-small-cell lung cancer (AUC, 0.89; 95% CI, 0.83–0.95; *p* < 0.001), independent of age of the individuals, smoking habits, whole-blood storage time, and various inflammatory conditions.
Łukasiewicz, Marta, et al. [30]	2021	Plasma, buffy coat	TEPs RNA + ctDNA	295 patients [279 TEPs RNA, 16 ctDNA]	Endometrial cancer (non-endometrioid and endometrioid, different stages)	Random forest [ctDNA] + deep neural network [TEPs RNA]	Platelet-dedicated classifier yielded AUC of 97.5% in the test set when discriminating between healthy subjects and cancer patients. However, the discrimination between endometrial cancer and benign gynecologic conditions was more challenging with AUC of 84.1%.
Zhang, Yu-Hang, et al. [31]	2017	Gene expression profiles (blood) *	TEPs mRNA	285 samples	Breast cancer, colorectal cancer, glioblastoma, hepatobiliary cancer, lung cancer, pancreatic cancer	Support vector machine incremental feature selection	Quantitative gene expression profiles were used to encode each sample. The results indicated that these genes could be important biomarkers for discriminating different cancer subtypes and healthy controls.
Fehlmann, Tobias, et al. [32]	2020	Blood	miRNA	3102 patients	Lung cancer (NSCLC and SCLC)	Gradient boosted trees (LightGBM)	First, a 15-miRNA signature from the training set was used to distinguish patients diagnosed with lung cancer from all other individuals in the validation set with an accuracy of 91.4% (95% CI, 91.0–91.9%), a sensitivity of 82.8% (95% CI, 81.5–84.1%), and a specificity of 93.5% (95% CI, 93.2–93.8%). Second, a 14-miRNA signature from the training set was used to distinguish patients with lung cancer from patients with nontumor lung diseases in the validation set with an accuracy of 92.5% (95% CI, 92.1–92.9%), sensitivity of 96.4% (95% CI, 95.9–96.9%), and specificity of 88.6% (95% CI, 88.1–89.2%). Third, a 14-miRNA signature from the training set was used to distinguish patients with early-stage lung cancer from all individuals without lung cancer in the validation set with an accuracy of 95.9% (95% CI, 95.7–96.2%), sensitivity of 76.3% (95% CI, 74.5–78.0%), and specificity of 97.5% (95% CI, 97.2–97.7%).
Yuan, Fei, et al. [33]	2021	Extracellular microRNA profiles *	miRNA	4046 samples	Benign ovarian disease, borderline ovarian tumor, breast cancer, colorectal cancer, esophageal cancer, gastric cancer, hepatocellular carcinoma, lung cancer, ovarian cancer, pancreatic cancer, sarcoma 12 classes, including 11 cancer types and 1 non-cancer class (2565 miRNAs used for each sample)	Random forest + support vector machine + k-nearest neighbor + decision tree	Feature selection and machine learning models with inherited information at the extracellular miRNA level were implemented to present a new workflow for cancer-classification recognition, early diagnosis, and monitoring with high prediction specificity. Selected microRNAs were then evaluated using the maximum relevance minimum redundancy method (mRMR), resulting in a feature list that was fed into the incremental feature selection method to identify candidate circulating extracellular microRNA for cancer recognition and classification. A series of quantitative classification rules was also established for such cancer classification.

* profiles downloaded from Gene Expression Omnibus.

## Data Availability

Not applicable.
